# Utilization of Inpatient Mental Health Care in the Rhineland During the COVID-19 Pandemic

**DOI:** 10.3389/fpubh.2021.593307

**Published:** 2021-04-30

**Authors:** Jürgen Zielasek, Jürgen Vrinssen, Euphrosyne Gouzoulis-Mayfrank

**Affiliations:** ^1^LVR-Institut für Versorgungsforschung, Köln, Germany; ^2^LVR-Klinik Köln, Köln, Germany

**Keywords:** in-patient and day patient mental healthcare, utilization, COVID-19, time series, pandemic

## Abstract

**Background:** During the Coronavirus-19 (COVID-19) pandemic, considerable changes occurred in the utilization of mental health care.

**Objectives:** We conducted an analysis of the changes of inpatient and day patient mental health care utilization in an association of psychiatric hospitals during the COVID-19 pandemic.

**Materials and Methods:** We used the statistics database of the association of nine psychiatric hospitals of the Rhineland Regional Council (Landschaftsverband Rheinland, LVR). We compared the case numbers of spring 2019 and spring 2020 and analyzed alterations in the diagnostic spectrum. Finally, we analyzed the age, gender, and diagnoses of patients tested positive for COVID-19.

**Results:** A total of 25,612 inpatient psychiatric hospital admissions were assessed. Case rates decreased by 25% during the COVID-19 pandemic. Changes varied between diagnostic groups, and there were even increases in case numbers for certain diagnoses. Women and patients of higher ages were overrepresented among psychiatric inpatients with COVID-19.

**Conclusions:** The COVID-19 pandemic resulted in considerable reductions in the total number of mental health-care admissions and in changes in the diagnostic spectrum. The results may be explainable by deferrals of elective hospital admissions during the acute phase of the pandemic and by destabilizing effects of the pandemic and social distancing on people with mental disorders.

## Introduction

The Corona virus-19 disorder (COVID-19) pandemic developed rapidly worldwide in early 2020 (1), and health-care systems had to be reorganized in order to be able to cope with large numbers of critically ill COVID-19 patients. During the same period, reductions in hospital admissions for other somatic conditions and reductions in emergency admissions were noted (2, 3). Until now, there have been few reports about the utilization of inpatient mental health care during the COVID-19 pandemic.

During the first wave of the pandemic, Tromans and coworkers reported an approximate reduction in referrals to mental health services of about 38% in the National Health Service of Leicestershire United Kingdom (4). Another study from Leicestershire showed that the number of inpatient admissions was ~20% lower during a 4-week period starting in mid-March 2020 compared to the same periods in 2018 and 2019 (5). Inpatient numbers were shown to be decreased due to reduced admissions and have a spike in discharges with the lockdown in the Cambridgeshire & Peterborough National Health Services Trust (6). Finally, emergency mental health service use decreased by 27% in the Central Institute for Mental Health in Mannheim, Germany (7).

Some non-peer-reviewed analyses reported further indicators of reduced utilization of psychiatric services. In the South London and Maudsley area National Health Service, Stewart and coworkers showed relatively stable caseloads and daily contacts in community mental health treatment, but some reductions in home treatment services and a shift to video consultations (8). In addition, they reported a 26% reduction in days as inpatients during the 31-day period after mid-March compared to the 31-day period before mid-March 2020. When comparing the same periods in 2019, they found a much lower reduction of only 3–8% (9). Finally, inpatient numbers decreased by 13.6% in the time period March 16–March 30, 2020, compared to the time period February 1–March 15, 2020 (10). Primary-care psychological therapies were even more affected: the number of patients accessing psychological therapies for anxiety and depression in southern England dropped by an average of 55% in the 9 weeks after lockdown (11).

Health-care services prepared themselves for the expected increased number of COVID-19 patients, and this included psychiatric hospitals (12). Mental health-care processes in inpatient services were restructured and reorganized, for example, by reorganizing regular psychiatric wards into COVID-19 isolation wards. Hygiene measures led to reductions of inpatient group therapies, protective face masks limited personal contact, and restrictions on visitors in hospitals due to inpatient lockdown regulations further reduced social contacts for inpatients [see (13) for examples from a German psychiatric hospital and Richter and Zürcher (14) for a more general discussion].

These changes also occurred in the nine psychiatric hospitals of the hospital association of the Rhineland Regional Council [Landschaftsverband Rheinland (LVR)]. These hospitals with ~2,835 beds and 750 day patients places provide mental health-care services to approximately half of the population of the Rhineland (4.4 million inhabitants). Analyzing the inpatient utilization rates during the acute phase of the COVID-19 pandemic of such a large population may provide an important empiric foundation for preparing psychiatric hospitals for future virus pandemics. Notably, the actual average Day Mix Index (DMI) (reflects the ratio of case severity and duration of inpatient treatment) of the nine LVR hospitals is 0.9687, which is comparable to the DMI of 0.9613 of 74 psychiatric hospitals and departments in Northrhine Westphalia (NRW), Bavaria, and Saxony Anhalt (source: Krankenhauszweckverband, LVR Clinics Association). We analyzed the daily inpatient and day-patient admission rates and the clinical characteristics of the psychiatric inpatient COVID-19 cases of the nine participating psychiatric hospitals in the spring of 2020, during the first wave of the COVID-19 pandemic in Germany.

## Method

The study was performed in accordance with the principles of the Helsinki Declaration. We analyzed the routine data of all inpatient and day-patient admission cases in all departments of the nine psychiatric hospitals of the LVR (general psychiatry, child and adolescent psychiatry, psychosomatic medicine, geriatric psychiatry, and addiction psychiatry) during the reporting periods from March 18 until May 31 for the years 2019 and 2020. The data included both planned and emergency admissions. Out-patient cases were not analyzed. Missing values of important data such as age or gender were very rare, and therefore no imputation of missing values was necessary.

To assess if the numbers of daily inpatient admissions were different before and during the COVID pandemic, we compared the daily admission numbers in the reporting periods of the years 2019 and 2020 using the Wilcoxon matched-pairs signed-rank test (double sided). To study if the COVID pandemic changed the diagnostic spectrum of inpatient cases, we assessed the frequencies of the main psychiatric diagnostic groups upon inpatient discharge and compared them before and during the COVID pandemic using the Chi-Square test. We did not use the admission diagnoses because these frequently change during the hospital stay due to incoming diagnostic information. In addition, we compared the mean age of patients who tested positive for COVID with the mean age of the non-COVID patients using the Student's *t*-test. Finally, we compared the gender distribution between COVID- and non-COVID patients using the Chi-Square test. We used a cutoff of *p* < 0.01 to indicate statistical significance. We used standard statistical software (GraphPad Prism version 9.0.0).

Routine data of the nine psychiatric hospitals of the Rhineland Regional Council are transferred on a daily basis to an anonymized statistics database, which serves quality assurance purposes and for the further development of quality of care. For the purpose of this study, we analyzed the aggregated data of all psychiatric departments of the nine hospitals (time series analysis of the sum of daily inpatient and day-patient admission rates, frequency of diagnoses of mental disorders, gender, and mean age of patients). Excel tables and arithmetic functions provided by Excel were used.

## Results

**Figure 1:**
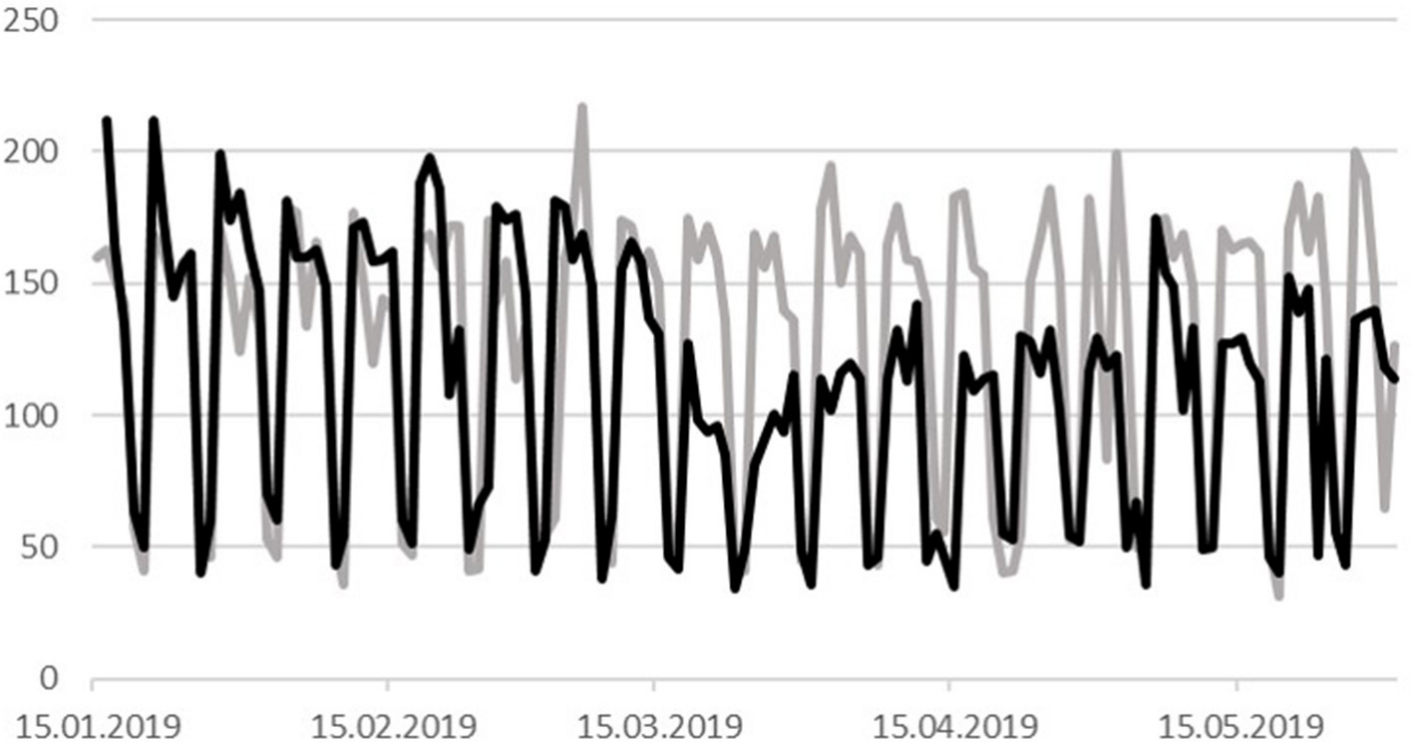
Daily inpatient/day-patient admission rates in the time period January 15–May 31, 2019 (gray line), and the corresponding time period of the year 2020 (black line). The 2020 time series was adjusted to corresponding weekdays of 2019. In addition, an intercalary day (February 29, 2020) was considered. Weekly reductions in admission rates are due to weekend days.

**Figure 2:**
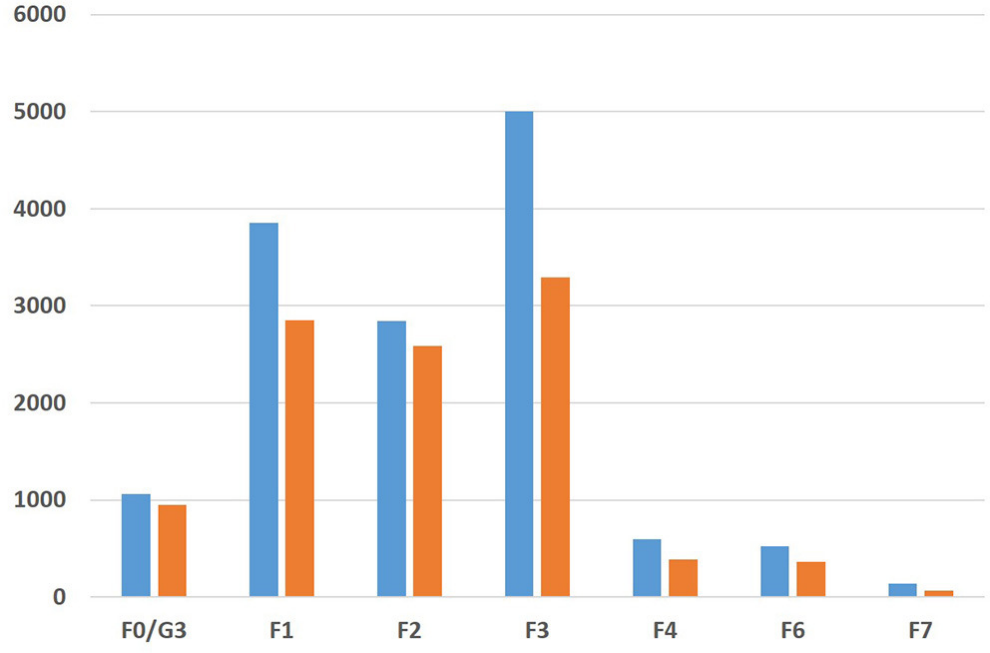
Numbers of inpatients/day patients of the nine psychiatric hospitals of the hospital association of the Rhineland Regional Council in the time period March 18–May 31 in the years 2019 (blue bars) and 2020 (orange bars). Given are the codes of the tenth revision of the International Classification of Disorders of the World Health Organization (ICD-10). F0/G3: Organic, including symptomatic, mental disorders, and Alzheimer's disease. F1: Mental and behavioral disorders due to psychoactive substance use. F2: Schizophrenia, schizotypal, and delusional disorders. F3: Affective disorders. F4: Neurotic, stress-related, and somatoform disorders. F6: Disorders of adult personality and behavior. F7: Mental retardation. ICD-10 groups F5 (behavioral syndromes associated with physiological disturbances and physical factors). F8: Disorders of psychological development and F9: Behavioral and emotional disorders with onset usually occurring in childhood and adolescence presented with <10 cases and are not shown.

During the course of the COVID-19 pandemic, daily admission rates decreased in the time period March 18–May 31, 2020 (*n* = 10,545), by 25% compared to the same time period of the previous year (*n* = 14.067) (Figure 1). The daily admission rates were significantly different between both years (Wilcoxon matched-pairs signed-rank test, *p* < 0.0003). The admission rates of patients from different diagnostic groups according to the mental disorders section of the World Health Organization International Classification of Disorders (10th revision, ICD-10) decreased to varying degrees, and the overall frequencies of the 10 diagnostic groups (F0–F9) were significantly different in the 2 years of observation (Chi-Square test, df = 9, *p* < 0.0001). We found the most pronounced decreases in the diagnostic groups F7 (mental retardation: from 139 cases in 2019 to 68 cases in 2020, a reduction of 51%), F4 (neurotic, stress-related, and somatoform disorders: −35% from 594 to 385 cases), F3 (affective disorders: −34% from 5,002 to 3,288 cases), and F6 (disorders of adult personality and behavior: −31% from 525 to 363 cases). Compared to this, reductions of case numbers of the diagnostic groups F0/G3 (organic, including symptomatic, mental disorders and Alzheimer's disease) and F2 (schizophrenia, schizotypal, and delusional disorders) were less robust (−10% from 1,058 to 948 cases, and −9% from 2,844 to 2,586 cases, resp.) (Figure 2).

We analyzed the ten most frequent disorders of the year 2019 (same time period: March 18–May 31) and found the most pronounced reductions for cases with a moderate depressive episode (ICD-10 F32.1, −49% compared to 2019), mental and behavioral disorders due to use of opioids, dependency syndrome (F11.2, −43%), and severe depressive episode without psychotic symptoms (F32.2, −41%) (Table 1A).

**Table 1A:** Diagnosis-specific case numbers in the observation period March 18–May 31 of the years 2019 and 2020 in the nine psychiatric hospitals.

**Diagnosis** **ICD-10 code**	**Text**	**Numbers of ** **cases 2019**	**Numbers of** **cases 2020**	**Relative** **change (%)**
F10.2	Mental and behavioral disorders due to use of alcohol, dependence syndrome	2,085	1,577	−24
F33.2	Recurrent depressive disorder, current episode severe without psychotic symptoms	1,921	1,324	−31
F20.0	Paranoid Schizophrenia	1,860	1,645	−12
F32.2	Severe depressive episode with psychotic symptoms	1,556	922	−41
F11.2	Mental and behavioral disorders due to use of opioids, dependence syndrome	712	403	−43
F05.1	Delirium superimposed on dementia	427	414	−3
F33.1	Recurrent depressive disorder, current episode moderate	403	248	−38
F60.31	Emotionally unstable personality disorder, borderline	395	291	−26
F32.1	Moderate depressive episode	313	161	−49
F43.2	Adjustment disorder	277	191	−31

*Shown are the 10 most-frequent diagnoses during the observation period in 2019 and the change from 2019 to 2020. ICD-10: 10th revision of the International Classification of Disorders issued by the World Health Organization*.

**Figure 3:**
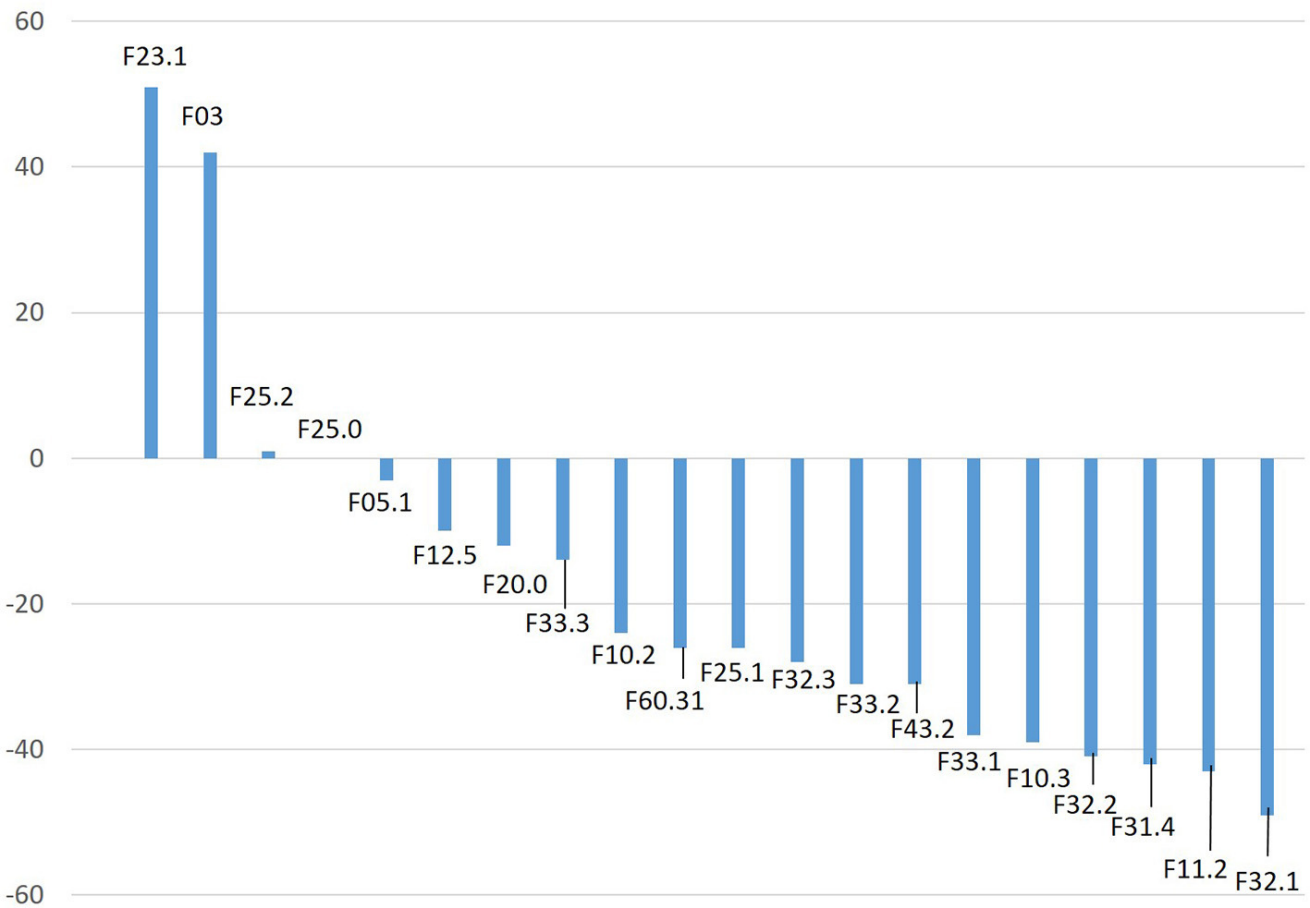
Changes of the case numbers of the 20 most frequent diagnoses in the observation period March 18–May 31, 2019. Diagnoses are ranked by the degree of relative change from 2019 to 2020 (% of case numbers of 2019). Codes as of the 10th revision of the International Classification of Disorders issued by the World Health Organization.

When analyzing the 50 mental disorders, which occurred in at least 20 cases during the time period of March 18–May 31, 2019, we found a decrease of cases in 38 disorders, near constancy in 3 disorders, and increases in 9 disorders. We found the greatest increase of case numbers in patients with an acute polymorphic psychotic disorder with symptoms of schizophrenia (F23.1, +51%: from 82 cases in 2019 to 124 cases in 2020), mental and behavioral disorders due to multiple drug use and use of other psychoactive substances, dependence syndrome (F19.2, +50%: from 42 cases in 2019 to 63 cases in 2020), and mental and behavioral disorders due to use of alcohol, acute intoxication (F10.0, +44%: from 48 cases in 2019 to 69 cases in 2020). Figure 3 and Table 1B show the ranks of changes during the reporting period from 2019 to 2020 of the 20 most frequent mental disorders of 2019. F19.2 und F10.0 do not appear here since they were not among the 20 most frequent disorders of the year 2019.

**Table 1B:** Diagnosis-specific case numbers in the observation period March 18–May 31 of the years 2019 and 2010 in the nine psychiatric hospitals.

**Rank**	**Diagnosis** **ICD-10 code**	**Text**	**Number of** **cases 2019**	**Number of** **cases 2020**	**Relative** **change (%)**
1	F23.1	Acute polymorphic psychotic disorder with symptoms of schizophrenia	82	124	+51
2	F03	Unspecified dementia	73	104	+42
3	F25.2	Schizoaffective disorder, mixed type	172	174	+1
4	F25.0	Schizoaffective disorder, manic type	201	202	0
5	F05.1	Delirium superimposed on dementia	427	414	−3
6	F12.5	Mental and behavioral disorders due to use of cannabinoids, psychotic disorder	136	122	−10
7	F20.0	Paranoid schizophrenia	1,860	1,645	−12
8	F33.3	Recurrent depressive disorder, current episode severe with psychotic symptoms	228	197	−14
9	F10.2	Mental and behavioral disorders due to use of alcohol, dependence syndrome	2,085	1,577	−24
10	F60.31	Emotionally unstable personality disorder, borderline	395	291	−26
11	F25.1	Schizoaffective disorder, depressive type	265	195	−26
12	F32.3	Severe depressive episode with psychotic symptoms	179	129	−28
13	F33.2	Recurrent depressive disorder, current episode severe without psychotic symptoms	1,921	1,324	−31
14	F43.2	Adjustment disorders	277	191	−31
15	F33.1	Recurrent depressive disorder, current episode moderate	403	248	−38
16	F10.3	Mental and behavioral disorders due to use of alcohol, withdrawal state	268	164	−39
17	F32.2	Severe depressive episode without psychotic symptoms	1,556	922	−41
18	F31.4	Bipolar affective disorder, current episode severe depression without psychotic symptoms	153	89	−42
19	F11.2	Mental and behavioral disorders due to use of opioids, dependence syndrome	712	403	−43
20	F32.1	Moderate depressive episode	313	161	−49

*Shown are the 20 most-frequent diagnoses of the observation time period of the year 2019 sorted by their ranks of relative change from 2019 to 2020. ICD-10: 10th revision of the International Classification of Disorders issued by the World Health Organization*.

During the reporting period, 28 patients with COVID-19 were documented among inpatients of the nine psychiatric hospitals. The mental disorders diagnosed in these patients were organic mental disorders including Alzheimer's dementia (*n* = 11), affective disorders (*n* = 10), schizophrenia (*n* = 4), substance use disorders (*n* = 2), and adjustment disorders (*n* = 1). Most cases occurred in women (75%), while women constituted only 51% of all 8,696 inpatients and day patients during the reporting period (Chi Square test, df = 1, *p* < 0.0015). Patients with COVID-19 were significantly older than the patients without COVID-19 [mean age (±standard deviation) 67.5 (±19.9) years vs. 48.3 (±18.3) years; Student's *t*-test *p* < 0.001]. This finding corresponded with an overrepresentation of organic mental disorders among the patients who tested positive for COVID-19: 39% of all psychiatric patients with COVID-19 were diagnosed with an organic mental disorder including Alzheimer's dementia, while this was the case in only 9% of the psychiatric non-COVID-19 cases. For affective disorders, this relationship was more balanced: 36% of the COVID-19 patients and 33% of the non-COVID-19 cases were diagnosed with an affective disorder (F3). In other diagnostic groups, the numbers of patients with COVID-19 were four or fewer; therefore, we cannot judge whether the distribution among COVID-19 and non-COVID-19 cases was balanced or not.

## Discussion

Our analyses show that the first wave of the COVID-19 pandemic was associated with considerable changes in the utilization of inpatient/day-patient services of a large psychiatric hospital association in the German Rhineland region. The region has both rural and urban parts, including the major metropolitan regions of Cologne, Düsseldorf, and the western part of the Ruhr Valley. Our analysis is limited by the regional constraint. However, with more than four million inhabitants served by the LVR Clinics Association, we provide the first analysis of the full data of one of the largest providers of psychiatric inpatient and outpatient hospital care in Germany. Until the end of the observation period, which included the end of the first wave of COVID-19 in Germany, admission rates remained lower than in the corresponding time period of the previous year. This finding implies considerable effects on the provision of mental health care for people with mental disorders. While the results of our study cannot be generalized to other regions and countries, another study in Germany showed a ca. 27% decline in emergency mental-health service use in one psychiatric hospital, which is comparable with our results, and evidence from studies from the UK and Germany points to the same direction of an overall reduction of inpatient mental healthcare utilization (7–11). Our study adds new information about a diagnostic shift toward more acutely ill patients. Of course, the data do not allow an assessment of whether the change in utilization behavior was associated with diagnostic delay or a shift of inpatient cases to later disease stages or with poorer treatment outcomes due to delayed interventions.

The long-term effects of the COVID pandemic on inpatient mental health-care utilization remain to be seen, especially as experts predicted long-term increases of mental-health problems due to COVID-19-associated psychological burden and social contact restrictions (15) with patients with preexisting mental disorders being at increased risk (16). Therefore, our findings from the first wave of the COVID pandemic may not be predictive for mental health-care utilization in the near future (17, 18). This corresponds to findings from earlier Coronavirus-associated pandemics demonstrating a long-lasting increase of mental disorders (19) and an associated increase of mental health-care utilization (20). In addition, at the time of writing of this report in October 2020, a second wave of COVID-19 cases in Germany became apparent.

In our study, admission rates declined more clearly in groups of patients with disorders which frequently led to elective (non-emergency) mental hospital admission (depressive disorders and admission for detoxication from opioid dependency). This profound decline may be the result of delaying planned inpatient admissions due to the provisioning of capacities for COVID-19 cases and other hygiene-related measures. On the other hand, admission rates increased for patient groups with disorders which frequently led to emergency admissions, such as acute psychotic disorders and alcohol intoxication. This finding may reflect the destabilizing effects of the pandemic and the social lockdown regulations on pre-existing mental disorders. This assumption is corroborated by early case reports about the exacerbation of psychotic disorders during the COVID-19 pandemic (16, 21). Initial reports also indicate that the use of substances of addiction was changing in Germany during the COVID-19 pandemic (22). It remains to be seen how the altered patterns of mental health-care utilization will change in the near future. The incipient second COVID-19 wave may become superimposed on urgent admissions due to previous delays of admissions (18), and this may result in a total increase in case numbers. Mental health-care services will definitely change and empirical data as in our study may help to inform these processes (23). Further analyses are warranted to examine the potential effects of sociodemographic and clinical variables on inpatient psychiatric services utilization during times of virus pandemic.

The findings of a preponderance of women, people of higher ages, and patients with organic mental disorders among the psychiatric cases of COVID-19 must be interpreted with caution due to the low numbers of cases. Interestingly, a recent analysis of electronic health records in the US showed an increased risk of COVID-19 in people with mental disorders, and, similarly to the results of our study, the risk was especially increased for women and for people of higher ages (24). Further long-term studies and studies in other regions and care settings are needed to address this issue. The findings may reflect a real increase of COVID-19 cases among these patient groups, or they may be due to a positive testing bias in high-risk groups of severe courses of COVID-19 and in women.

The total number of patients with COVID-19 among patients of the nine psychiatric hospitals was rather low during this initial wave of the pandemic. Long-term studies are now warranted since increases in case numbers of COVID-19 are incipient, and the abovementioned mechanisms may lead to overall increased utilization of inpatient psychiatric hospital services in the future (18).

## Data Availability Statement

The data analyzed in this study is subject to the following licenses/restrictions: anonymised and aggregated raw data are made available upon due request to reproduce the statistical analyses. Requests to access these datasets should be directed to juergen.zielasek@lvr.de.

## Ethics Statement

Ethical review and approval was not required for the study on human participants in accordance with the local legislation and institutional requirements. Written informed consent for participation was not required for this study in accordance with the national legislation and the institutional requirements.

## Author Contributions

JZ, JV, and EG-M: conception and design, data collection, analysis and interpretation of the data, drafting the article, revising the article critically for important intellectual content, and final approval of the version to be published. All authors contributed to the article and approved the submitted version.

## Conflict of Interest

The authors declare that the research was conducted in the absence of any commercial or financial relationships that could be construed as a potential conflict of interest.
